# A pan-cancer study of the transcriptional regulation of uricogenesis in human tumours: pathological and pharmacological correlates

**DOI:** 10.1042/BSR20171716

**Published:** 2018-09-19

**Authors:** Zuzana Saidak, Christophe Louandre, Samy Dahmani, Chloé Sauzay, Sara Guedda, Bruno Chauffert, Denis Chatelain, Irene Ceballos-Picot, Antoine Galmiche

**Affiliations:** 1 Equipe CHIMERE, Université de Picardie Jules Verne, Amiens, France; 2 Laboratoire d’Oncobiologie Moléculaire, Centre de Biologie Humaine, CHU Sud, Amiens, France; 3 Laboratoire de Biochimie, Centre de Biologie Humaine, CHU Sud, Amiens, France; 4 Service d’Oncologie Médicale, CHU Sud, Amiens, France; 5 Service d’Anatomopathologie, CHU Nord, Amiens, France; 6 Laboratoire de Biochimie métabolomique et protéomique, Hôpital Necker, Assistance Publique Hôpitaux de Paris, Paris, France

**Keywords:** APRT, 5-Fluorouracile, TCGA, uric acid, XDH

## Abstract

Uric acid (UA) is the end product of the catabolism of purines, and its serum levels are commonly increased in cancer patients. We aimed to explore the transcriptional regulation of tumour uricogenesis in human tumours, and relate uricogenesis with tumour pathological and pharmacological findings. Using data from The Cancer Genome Atlas (TCGA), we analysed the expression levels of xanthine dehydrogenase (XDH) and adenine phosphoribosyltransferase (APRT), two key enzymes in UA production and the purine salvage pathway, respectively. We found large differences between tumour types and individual tumours in their expression of *XDH* and *APRT*. Variations in locus-specific DNA methylation and gene copy number correlated with the expression levels of *XDH* and *APRT* in human tumours respectively. We explored the consequences of this differential regulation of uricogenesis. Tumours with high levels of *XDH* mRNA were characterised by higher expression of several genes encoding pro-inflammatory and immune cytokines, and increased levels of tumour infiltration with immune cells. Finally, we studied cancer drug sensitivity using data from the National Cancer Institute-60 (NCI-60) database. A specific correlation was found between the expression levels of *APRT* and cell sensitivity to the chemotherapeutic agent 5-fluorouracil (5-FU). Our findings underline the existence of great differences in uricogenesis between different types of human tumours. The study of uricogenesis offers promising perspectives for the identification of clinically relevant molecular biomarkers and for tumour stratification in the therapeutic context.

## Introduction

**Figure 1:**
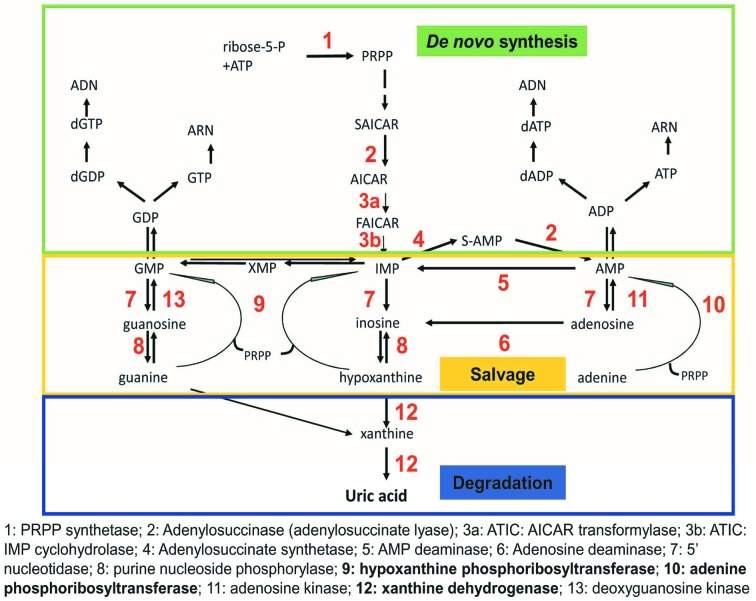
A summary of the cellular metabolism of purines Purines are synthesised by a multistep pathway known as *de novo* synthetic pathway. They are recycled via the salvage pathway, and ultimately are degraded and excreted as UA. We present the key steps in their metabolism towards UA. The three enzymes that are under investigation in the present study, i.e. APRT, HPRT and XDH, are highlighted here with bold characters.

Uric acid (UA) is the end product of the catabolism of purines. It is well-known for its ability to precipitate and form crystals, characteristic of gout, a sterile inflammatory syndrome of the joints [1–3]. It is also involved in the formation of renal calculi due to the high insolubility of UA in urine. Hyperuricemia, i.e. increased serum UA is associated with various common pathological conditions, such as diabetes and the metabolic syndrome [4,5]. Through recent research, it has emerged that purines and their final catabolic end-product are not only essential structural components of nucleic acids, but also messengers able to regulate multiple facets of cellular physiology [4,5]. The enzyme xanthine dehydrogenase/xanthine oxidase (*XDH*) catalyses the final steps of purine catabolism, by transforming hypoxanthine to xanthine, and subsequently to UA. It is usually considered as the rate-limiting step for the synthesis of UA [4]. Conversely, the rate of UA synthesis is limited by the existence of a purine salvage pathway, catalysed by the enzymes hypoxanthine phosphoribosyltransferase (HPRT, gene *HPRT1*) and adenine phosphoribosyltransferase (APRT, *APRT*). This salvage pathway permits the recycling of hypoxanthine guanine and adenine and limits their availability as substrates for uricogenesis (Figure 1).

Metabolic reprogramming is emerging as an essential step during malignant cell transformation [6,7]. Increased serum UA levels are commonly found in cancer patients [8]. High cellular turnover and the occurrence of tumour cell lysis are classically considered as an explanation for the increased UA levels in cancer patients. High serum UA levels are associated with aggressiveness in tumours originating from the colon and rectum [9,10], pancreas [11], breast [12] and the upper aero-digestive tract [13,14]. However, an increase in serum UA levels can also occur in the early stages of tumorigenesis [15–17]. The interpretation of serum values of UA in cancer patients is complex because individual dietary habits and exposure to carcinogenic agents, such as tobacco, might increase serum UA levels independent of tumour metabolism [15–17]. The existence of tumour-intrinsic differences, dictated by the tissue of origin and heterogeneity within one tumour type, has until now been poorly addressed. Also, previous studies addressing the regulation of uricogenesis at the molecular level were mainly centred on the regulation of XDH [8]. Little is known about the regulation of the enzymes that regulate the purine salvage pathway in human tumours. Another important question pertains to the pathological and therapeutic consequences of the regulation of uricogenesis in tumour cells. UA produced by tumour cells potentially plays a pro-inflammatory role and could promote the recruitment of an immune infiltrate in various experimental models [1–3,18,19]. UA is also a potential determinant of cancer cell response to medical therapeutics. In a recent study, UA was found to modulate cancer cell sensitivity to the antimetabolite 5-fluorouracil (5-FU), one of the most commonly used anticancer drugs in the clinic [20]. Whether molecular analysis centred on the genes involved in uricogenesis could provide interesting parameters and novel biomarkers for patient stratification remains to be shown.

In the present study, we aimed to address the existence of biological differences in the regulation of uricogenesis between different tumour types, and also between individual tumours within each tumour type. We also aimed to address the existence of pathological and pharmacological particularities associated with tumour uricogenesis. To address the transcriptional regulation of uricogenesis at the pan-cancer level, we used data obtained by RNA sequencing from The Cancer Genome Atlas (TCGA). TCGA is an international research initiative offering access to genomic and epigenomic results for >11000 human tumour samples, representing 33 cancer types [21,22]. Data regarding the pharmacological sensitivity of human cancer cells were accessed separately through the National Cancer Institute-60 (NCI-60). The NCI-60 is an open resource that provides access to the ‘Omics’ profiling and pharmacological response analyses for 60 human cell lines established from nine common tumour types [23]. The NCI-60 makes it possible to study the molecular determinants that regulate the efficacy of anticancer drugs [24]. Our study highlights the essential differences that exist between different tumour types and between individual tumours within each tumour type in their regulation of uricogenesis.

## Materials and methods

### TCGA datasets

Patients’ clinical and pathological data and RNA-seq expression values (RNA SeqV2 RSEM), were retrieved from TCGA in May 2017, using the portal Cbioportal at: http://cbioportal.org [25,26]. Twenty-four tumour datasets were analysed representing a total of 10663 patients: Acute Myeloid Leukaemia (AML, *n*=200), Bladder urothelial carcinoma (*n*=413), Brain lower grade glioma (*n*=530), Breast invasive carcinoma (*n*=1105), Cervical cancer (*n*=309), Colorectal adenocarcinoma (*n*=633), Oesophageal carcinoma (*n*=186), Glioblastoma (*n*=604), Head and Neck Squamous Cell Carcinoma (HNSCC, *n*=530), Kidney Renal Clear Cell Carcinoma (Kidney RCC, *n*=538), Kidney Renal Papillary Cell Carcinoma (Kidney RPC, *n*=293), Liver Hepatocellular Carcinoma (HCC, *n*=442), Lung adenocarcinoma (*n*=522), Lung squamous cell carcinoma (*n*=504), Ovarian serous cystadenocarcinoma (*n*=603), Pancreatic adenocarcinoma (PAAD, *n*=186), Prostate adenocarcinoma (*n*=499), Sarcoma (*n*=265), Skin cutaneous melanoma (*n*=479), Stomach adenocarcinoma (*n*=478), Testicular germ cell tumours (*n*=156), Thymoma (*n*=124), Thyroid carcinoma (*n*=516), Uterine corpus endometrial carcinoma (*n*=548). The tumour samples in TCGA are surgical resection samples obtained from primary tumours that have received no prior neoadjuvant treatment. For inter-tumour/pan-tumour analyses, gene expression was normalised to *TBP* (TATA-box binding protein). *Z*-score values were used when only one tumour type was studied.

### Tumour microenvironment analysis

The ESTIMATE immune score was used to analyse the infiltration levels of immune cells in different tumours. This analysis was based on the interpretation of gene expression profiles retrieved from TCGA expression data (http://bioinformatics.mdanderson.org/estimate/) [27]. The Microenvironment Cell Population-counter (MCP counter) method was used to quantify the absolute abundance of immune and stromal cell populations in tumours [28]. The MCP counter analyses the presence of gene signatures for eight immune cell types, and fibroblasts and endothelial cells [28]. The corresponding scores are proportional to the number of the corresponding cells present in each sample, thus allowing comparisons between individual tumour samples [28].

### NCI-60 analysis

The NCI-60 database, which contains data on 60 different cancer cell lines from nine different types of tumours, was accessed using the CellMiner interface (https://discover.nci.nih.gov/cellminer/). Basal mRNA expression levels (*z* scores) and cell sensitivity data (GI50) were retrieved for the 60 cell lines [23,29–31].

### Statistical analyses

Wilcoxon test, Spearman and Pearson correlations were used as indicated and were all performed using R version 3.4.2 (https://www.r-project.org) (package Hmisc).

## Results

### Cancer-dependent expression of genes involved in the uricogenesis pathway

**Figure 2:**
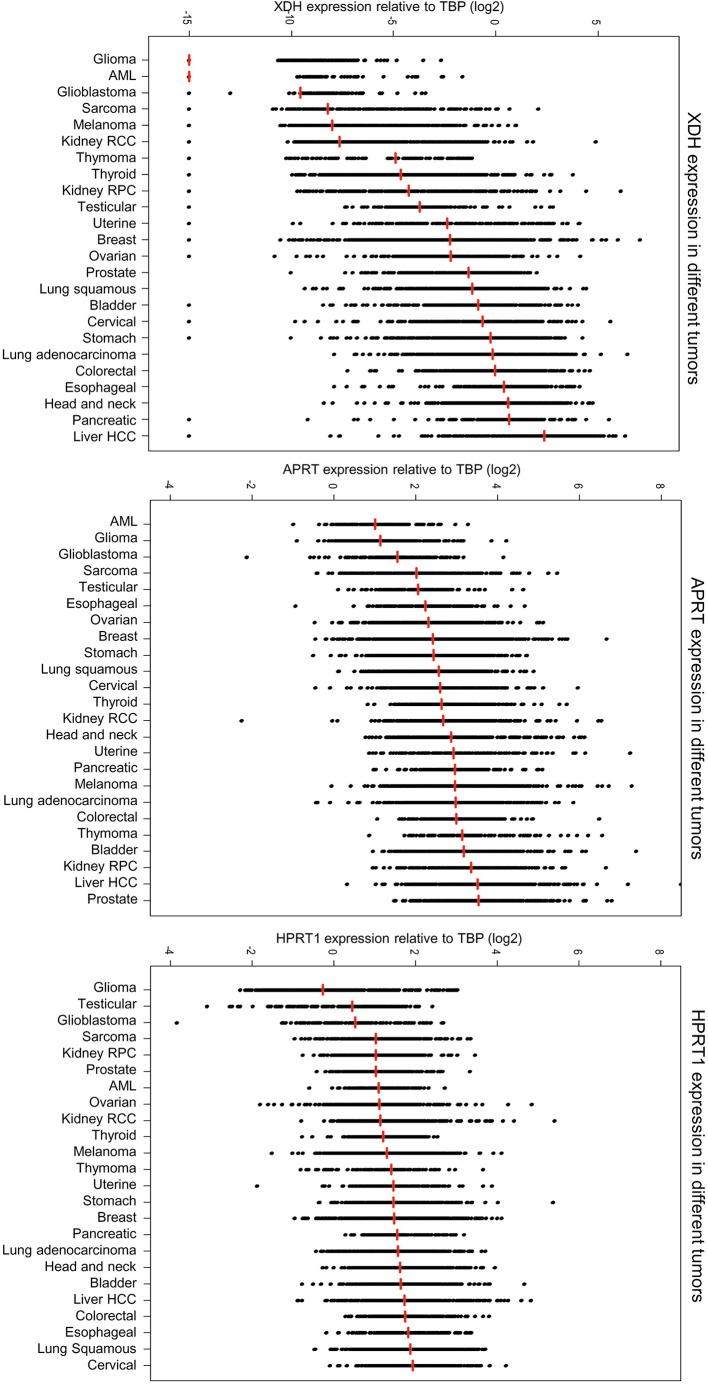
Expression of genes involved in uricogenesis is cancer type dependent A stripchart showing the mRNA expression levels of *XDH, APRT* and *HPRT1* in different primary tumours. Each point represents the expression of the gene in one tumour sample (log_2_(RNA Seq V2 RSEM normalised values relative to TBP)). Vertical lines indicate the median expression values.

To address the tumour-intrinsic regulation of uricogenesis, we examined the expression of the three genes encoding the core enzymes of uricogenesis, i.e. *APRT, HPRT1* and *XDH* (Figure 1). Data regarding the expression levels of the corresponding genes were extracted from TCGA and were compared among the different tumour types (Figure 2). We found a striking heterogeneity in the expression of these genes: median expression of the gene *XDH* showed the most striking heterogeneity with some tumours expressing negligible levels of *XDH* (for example, glioma and glioblastoma) while others were characterised by high *XDH* expression levels (PAAD, HCC and HNSCC). Interestingly, expression levels of *APRT* also showed great inter-tumour heterogeneity, yet to a smaller extent than *XDH* (a more than 10^2^-fold difference between the median expression values in the high compared with low-expressing tumour types) (Figure 2). These findings established the existence of intrinsic differences in uricogenesis between different tumour types and between individual tumours within each tumour type.

### Impact of tumour grade and identification of the genomic events involved in the regulation of uricogenesis

**Figure 3:**
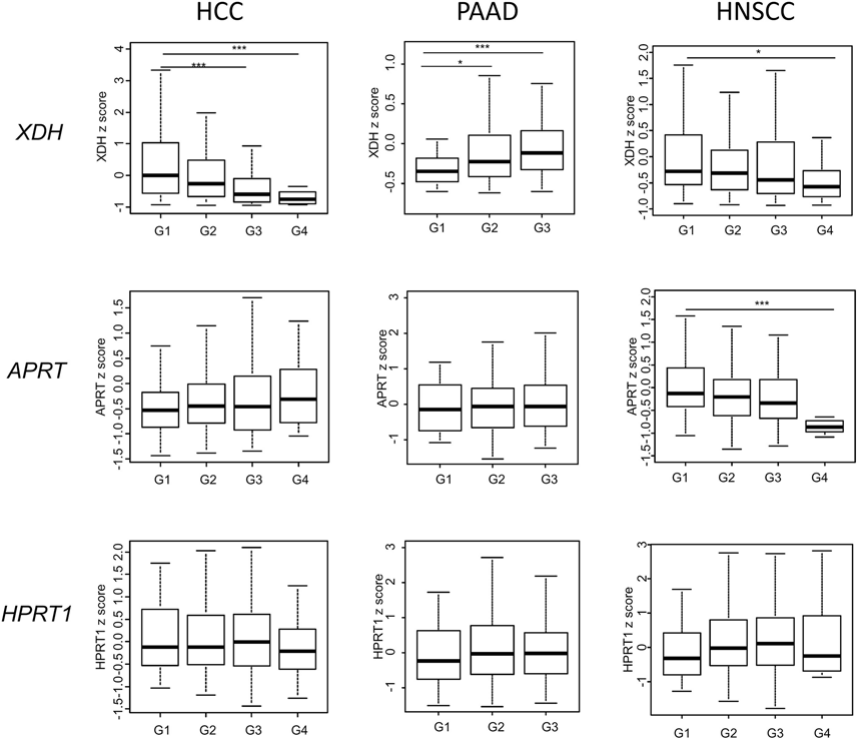
*XDH, APRT* or *HPRT1* expression according to the histological grade in HCC, PAAD and HNSCC The patients with HCC, PAAD and HNSCC were stratified according to their histological tumour grade, with G1 representing well-differentiated tumours and G3 poorly differentiated tumours. For HCC: G1 (*n*=55), G2 (*n*=180), G3 (n=124), G4 (*n*=13). For PAAD: G1 (*n*=24), G2 (*n*=94), G3 (*n*=47). For HNSCC: G1 (*n*=62), G2 (*n*=303), G3 (*n*=125), G4 (*n*=7). XDH was shown to be related to tumour grade in all three tumour types. *P<0.05, ***P<0.001.

To address the regulation of tumour uricogenesis, we attempted to correlate the expression levels of these genes with tumour characteristics in the three cancer types with the highest median *XDH* expression, i.e. PAAD, HNSCC and liver HCC. We found no significant association between patients’ age or gender and the expression levels of any of the genes involved in uricogenesis (data not shown). In PAAD, we found no significant difference in the expression of the genes *APRT, HPRT1* and *XDH* in tumours of different stage/extension to lymph nodes (data not shown). Tumour grade, however, significantly correlated with the expression levels of *XDH*: high-grade pancreatic tumours had significantly higher expression levels of *XDH* (G1 compared with G3, *P*=0.0006) (Figure 3). Compared with PAAD, an inverse pattern of expression was found in HCC and HNSCC. Lower levels of *XDH* gene expression were found in tumours of high grade (for HCC: G1 compared with G4, *P*=0.0021; for HNSCC: G1 compared with G4, *P*=0.0174) (Figure 3).

**Figure 4:**
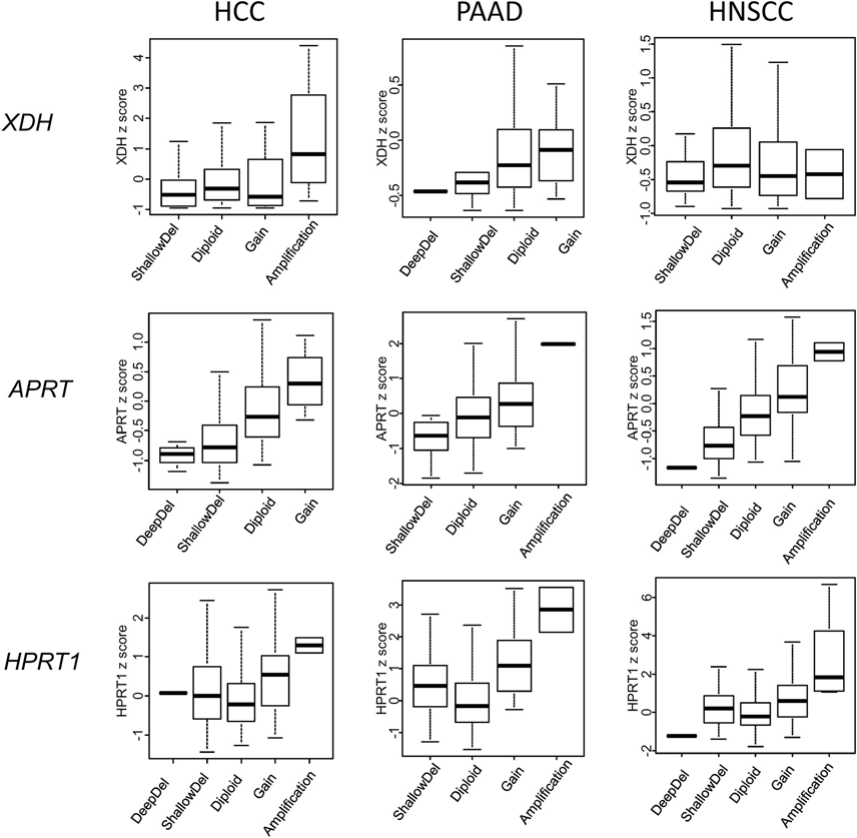
Uricogenesis gene expression and variations in gene copy number Analysis of the gene copy number for *XDH, APRT* and *HPRT1* in HCC (*n*=363), PAAD (*n*=168) and HNSCC (*n*=514) from TCGA. For each gene, the boxplots represent the gene expression levels according to the copy number, classified as deep deletion, shallow deletion, diploid, gain or amplification at the corresponding locus.

To address the mechanism through which cancer cells regulate expression levels of the enzymes involved in uricogenesis, correlation analyses were carried out for *XDH, HPRT1* and *APRT* mRNA levels compared with locus-specific DNA methylation/gene copy number variation. Using data from TCGA, *XDH* expression levels correlated either weakly (HCC, PAAD) or not (HNSCC) with gene copy number (Figure 3). For *XDH*, a better correlation was found between *XDH* mRNA levels and site-specific DNA methylation levels (Table 1): Spearman *R* = −0.62 for PAAD (*P*<1 × 10^−22^), *R* = −0.47 for HNSCC (*P*<1 × 10^−22^) and *R* = −0.36 for HCC (*P*=9.21 × 10^−13^). A distinct pattern of regulation was found for *APRT*. We observed variable gene copy number of *APRT* in individual tumours (defined as deep deletion, shallow deletion, diploid, gain or amplification) that significantly correlated with *APRT* mRNA levels (Figure 4). These findings suggest that the expression of each uricogenesis enzyme is under specific molecular regulation in cancer cells.

**Table 1:** Correlation analysis of *APRT* and *XDH* mRNA expression compared with DNA methylation in different tumours from TCGA

	APRT		XDH	
	*R*	*P*	*R*	*P*
HCC	−0.25	1.09 × 10^−6^	−0.36	9.21 × 10^−13^
PAAD	−0.40	3.61 × 10^−8^	−0.62	<1 × 10^−22^
HNSCC	−0.23	7.48 × 10^−8^	−0.47	<1 × 10^−22^
Colorectal	−0.22	1.67 × 10^−5^	−0.27	1.28 × 10^−7^
Lung adenocarcinoma	−0.18	0.0001	−0.13	0.0040
Bladder	−0.22	8.73 × 10^−6^	−0.37	2.22 × 10^−14^
Lung squamous	−0.21	4.59 × 10^−5^	−0.32	4.76 × 10^−10^
Prostate	−0.27	8.89 × 10^−10^	−0.33	4.35 × 10^−14^
Breast	−0.25	1.42 × 10^−12^	−0.14	6.70 × 10^−5^

Data shown are the correlation analyses between the gene expression and locus-specific DNA methylation. Spearman R and the *P*-value are indicated in each case. Note that no gene methylation data were available for *HPRT1*.

### Uricogenesis gene regulation as a determinant of tumour microenvironmental composition

**Figure 5:**
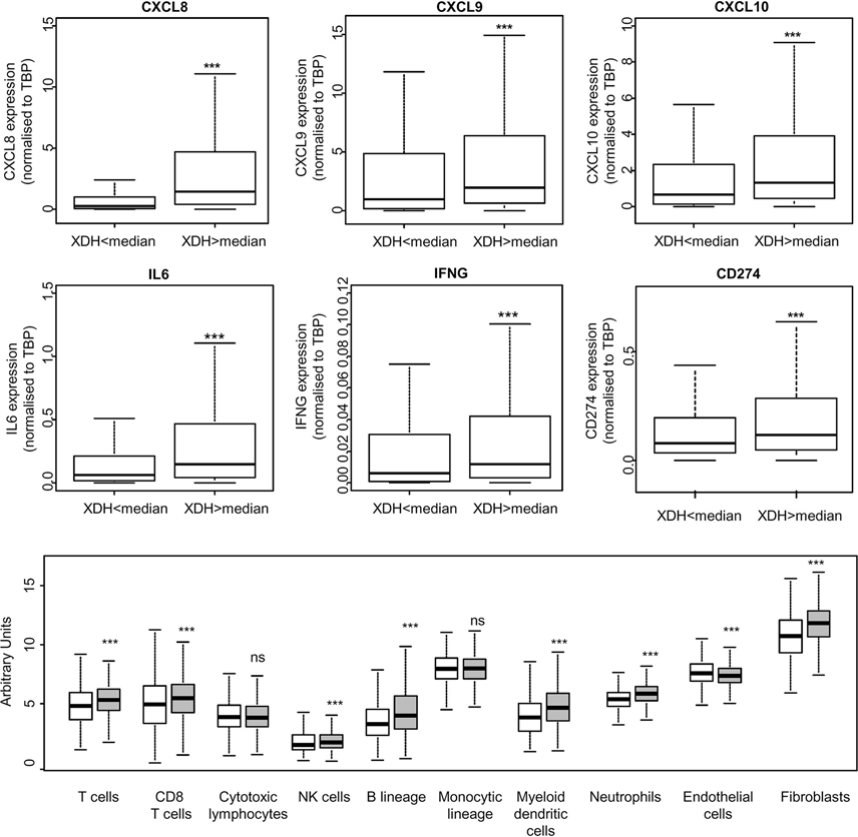
*XDH* expression is associated with elevated mRNA expression levels of pro-inflammatory and immune mediators Boxplots representing the expression of genes encoding inflammatory/immune cytokines. All available data for the 24 tumour types used in the present study was pooled (RNA Seq V2 RSEM normalised to TBP, *n*=9083). The data were stratified into two groups according to XDH levels, below or above the median. The abundance of immune and stromal cell populations in tumours was quantified using the MCP-counter method in tumours expressing low- or high-expression levels of XDH: <median (white) compared with >median (grey) using pooled data for 6195 tumours. The differential expression of the indicated immune markers or immune/stromal infiltrates was tested using the unpaired Wilcoxon test, ***P<0.001; ns, not significant.

In search of pathological parameters that would correlate with uricogenesis, we explored the expression levels of various biologically important inflammatory and immune cytokines in a pan-cancer manner. We analysed the expression levels of the chemokines with CXC motif ligands (CXCL): *CXCL8, CXCL9* and *CXCL10*, Interleukin-6 (*IL-6*), Interferon-γ (*IFNG*) as well as *CD274* (encoding the immune checkpoint regulator PD-L1, i.e. ligand of PD1) (Figure 5). For each gene, a comparison of the mRNA expression levels was carried out between two groups defined by the median value of *XDH*, normalised to the reference gene *TBP*. Using this approach, we found that tumours with higher expression of *XDH* have significantly higher mRNA levels of the genes encoding the corresponding immune/inflammatory regulators (Figure 5). We pursued our analysis by using the algorithm ESTIMATE, in order to assess the association of uricogenesis gene expression with the presence of infiltrating immune cells in the tumours [27]. We found significantly higher immune scores in tumours with high expression of *XDH:* median of 95.9 for tumours with low *XDH* compared with median of 230.6 for tumours with high *XDH* (*P*=1.6 × 10^−6^) indicating that higher tumour uricogenesis is associated with higher expression of various immune and pro-inflammatory cytokines and denser tumour immune infiltration.

The composition of the tumour microenvironment was addressed using the MCP counter method [28]. We observed that a number of immune cell types were present at higher levels in tumours with high *XDH* (*XDH* > median) compared with tumours with low *XDH* (*XDH* < median) in a pan-tumour analysis (*n*=6195 tumours) (Figure 5). This included T cells (*P*<2.2 × 10^−16^), CD8 T cells (*P*<2.2 × 10^−16^), NK cells (*P*=2.04 × 10^−12^), B-lineage cells (*P*<2.2 × 10^−16^) and myeloid dendritic cells (*P*<2.2 × 10^−16^). The greatest fold-increase in expression was seen for B lineage cells (1.22-fold increase in the median for *XDH* high compared with *XDH* low) and myeloid dendritic cells (1.22-fold increase in the median for *XDH* high compared with *XDH* low). Regarding stromal cells, fibroblasts were present at higher levels in high *XDH* tumours compared with low *XDH* tumours (*P*<2.2 × 10^−16^), whereas endothelial cells were present at significantly lower levels (*P*<2.2 × 10^−16^).

### Uricogenesis gene regulation as a determinant of cancer cell sensitivity to chemotherapy

**Figure 6:**
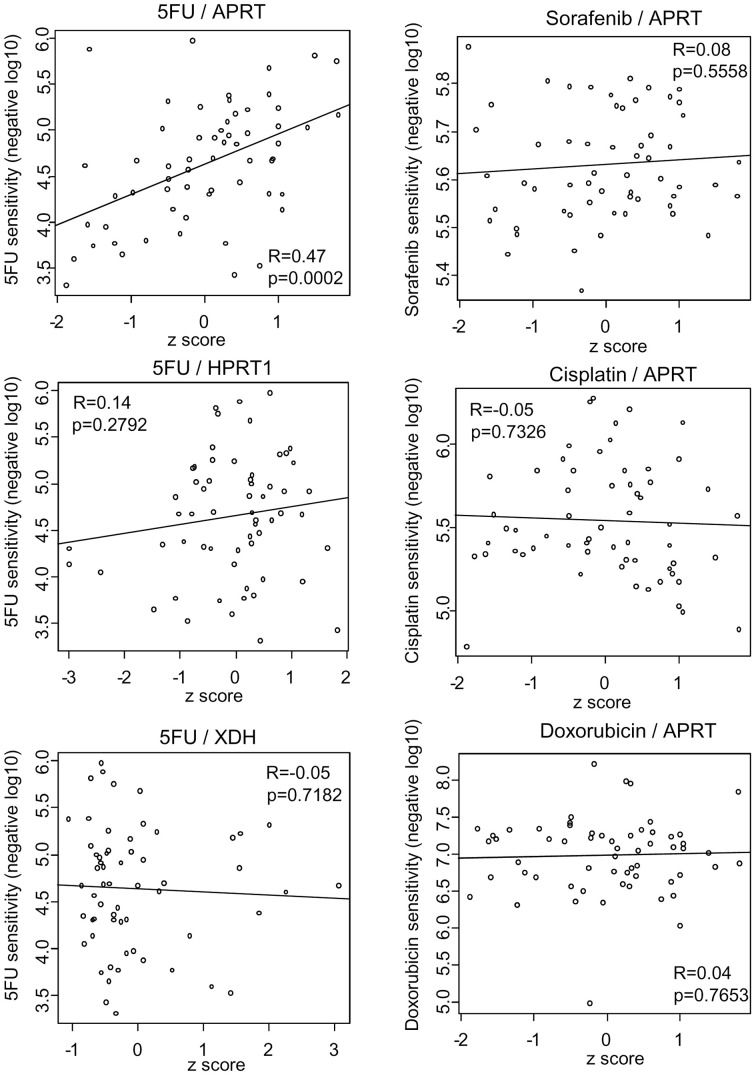
APRT is a potential determinant of 5-FU sensitivity in cancer cells Basal expression levels of the three genes involved in uricogenesis were correlated with drug sensitivity of the cancer cell lines from the NCI-60 panel. We present the correlation analysis between *APRT, HPRT1* and *XDH* gene expression and cancer cell sensitivity to 5-FU (Pearson R and *P*-value indicated each time) (left panel). No correlation was established between the expression of *APRT* and cancer cell sensitivity to other commonly used anticancer agents, such as sorafenib, cisplatin and doxorubicin (right panel).

To address the therapeutic implications of the transcriptional regulation of uricogenesis, we used the NCI-60 database. We observed different expression of the three key enzymes involved in uricogenesis among the 60 cell lines (Supplementary Figure S1). We systematically addressed the existence of a correlation between the expression levels of *APRT, HPRT1, XDH* in the 60 human cancer cell lines and their pattern of drug sensitivity (Figure 6). Interestingly, a statistically significant and positive correlation was found between the expression levels of *APRT* and the sensitivity of different cell lines to 5-FU (Pearson *R* = 0.47, *P*=0.0002). This observation was specific because cell sensitivity to 5-FU did not correlate with the expression levels of *XDH* or *HPRT1*. Conversely, the expression levels of *APRT* did not correlate with cell sensitivity to other anticancer therapies, such as sorafenib, cisplatin or doxorubicin (Figure 6). We concluded that the expression of *APRT* specifically correlates with tumour cell sensitivity to 5-FU.

## Discussion

It is increasingly accepted that metabolic reprogramming is central to malignant transformation, as was shown by past studies of cellular glucose or glutamine metabolism [6,7]. UA is a key metabolite and the end product of purine catabolism, but relatively little is known regarding the regulation of tumour uricogenesis compared with other cellular metabolic pathways [8]. Our study provides a first pan-cancer analysis of the regulation of tumour uricogenesis. We found great differences in gene expression patterns among different tumour types, and between individual patients within each tumour type. The differences were found to reside at the level of transcriptional regulation of the enzymes *XDH, APRT*, and to a lesser extent, *HPRT1*. The regulation of these enzymes is complex, with gene expression differentially regulated by copy number variation (*APRT*) or DNA methylation (*XDH*). We found an association between the expression levels of *XDH* and tumour expression of immune cytokines, as well as higher infiltration of immune and inflammatory cells in tumour tissues. Overall, these findings point to tumour uricogenesis as a potentially important biological parameter.

In addition to the observation that individual tumours vary greatly, even within each histological subtype/tissue of origin, our study highlights the particularities of some types of tumours. For example, glioblastoma were found to express extremely low levels of the mRNA encoding uricogenesis enzymes (in particular *XDH*). Glioblastoma exhibits many particularities in their intermediary metabolism, as was, for example underlined by studies of their lipid metabolism [32]. Our conclusion regarding the existence of particularities in UA metabolism in some types of tumours is based on the exploration of RNA expression levels and would require validation at the protein level. Nevertheless, the findings are supported by the study by Hu et al. [33] which suggests that the transcriptional regulation of the metabolic network of individual tumours closely resembles that of the primary tissue of origin.

Our study highlights *APRT*, in addition to *XDH*, as a source of individual variability in uricogenesis among tumours. APRT catalyses a salvage reaction and the recycling of adenine into AMP, thus limiting the need for *de novo* synthesis of purines by cancer cells. The expression levels of *APRT* were found to be variable among individual tumours, and they correlated with cancer cell sensitivity to 5-FU. We suggest that tumour cells that produce less UA as a consequence of increased APRT activity and purine salvage, might be more sensitive to 5-FU. These findings confirm and extend the results of Cantor et al. [20], who recently showed that the presence of UA in the cell culture medium limits the efficacy of 5-FU by directly inhibiting the enzyme UMP synthase [18]. We propose that *APRT* might be included in the list of genes that modulate cancer cell susceptibility to 5-FU [34–36]. Analysing the regulation of tumour uricogenesis might be of interest clinically for better prediction of the anticancer efficacy of 5-FU in cancer patients.

Another interesting observation that we report here is the existence of a strong and positive link between the levels of *XDH* and the expression levels of multiple genes encoding mediators of inflammation (*IL6, CXCL8, CXCL9, CXCL10*), the immune response (*IFNG* and *CD274*, encoding the immune checkpoint regulators PD-L1) and likely the tumour immune infiltrate itself. This finding is consistent with the results of past experimental studies that have shown that UA produced by tumour cells can play a pro-inflammatory and immune stimulatory role [1–3,18,19]. Interestingly, new medically approved therapeutics targeting the adaptive immune system have recently led to an improvement in the clinical outcome for various types of cancer in humans [37]. Because the overall efficacy of the corresponding immune checkpoint inhibitory drugs is limited to a subset of patients, an essential aim of medical research is to predict the benefit gained from these drugs by individual patients [37,38]. Recent studies show that tumour cell-intrinsic factors play an essential role in immune cell infiltration and the response to these therapies [39]. Based on our results obtained from a large panel of human tumours, we propose that analysing tumour uricogenesis may help predict the efficacy of immune checkpoint modulators in cancer patients.

## Supporting information

**Suppl. Fig. 1:** ***APRT, HPRT1* or *XDH* expression in different cell lines of the NCI-60 panel.** We present the transcript intensity for the APRT, HPRT1 or XDH genes, expressed as z score.

## Abbreviations

APRT: adenine phosphoribosyltransferase
CXCL: CXC motif ligand
HCC: hepatocellular carcinoma
HNSCC: head and neck squamous cell carcinoma
HPRT: hypoxanthine phosphoribosyltransferase
IFNG: interferon-γ
IL-6: interleukin-6
MCP-counter: microenvironment cell population-counter
NCI-60: National Cancer Institute-60
PAAD: pancreatic adenocarcinoma
TBP: TATA-box binding protein
TCGA: The Cancer Genome Atlas
UA: uric acid
XDH: xanthine dehydrogenase
5-FU: 5-fluorouracil
