# Supplementary material for: A pan-cancer study of the transcriptional regulation of uricogenesis in human tumours: pathological and pharmacological correlates
Source: Biosci Rep. 2018 Sep 19;38(5):BSR20171716. doi: 10.1042/BSR20171716 (PMC6146287; doi:10.1042/BSR20171716)
Supplement: Supplementary file 1 [file bsr20171716_Supp1.pdf]

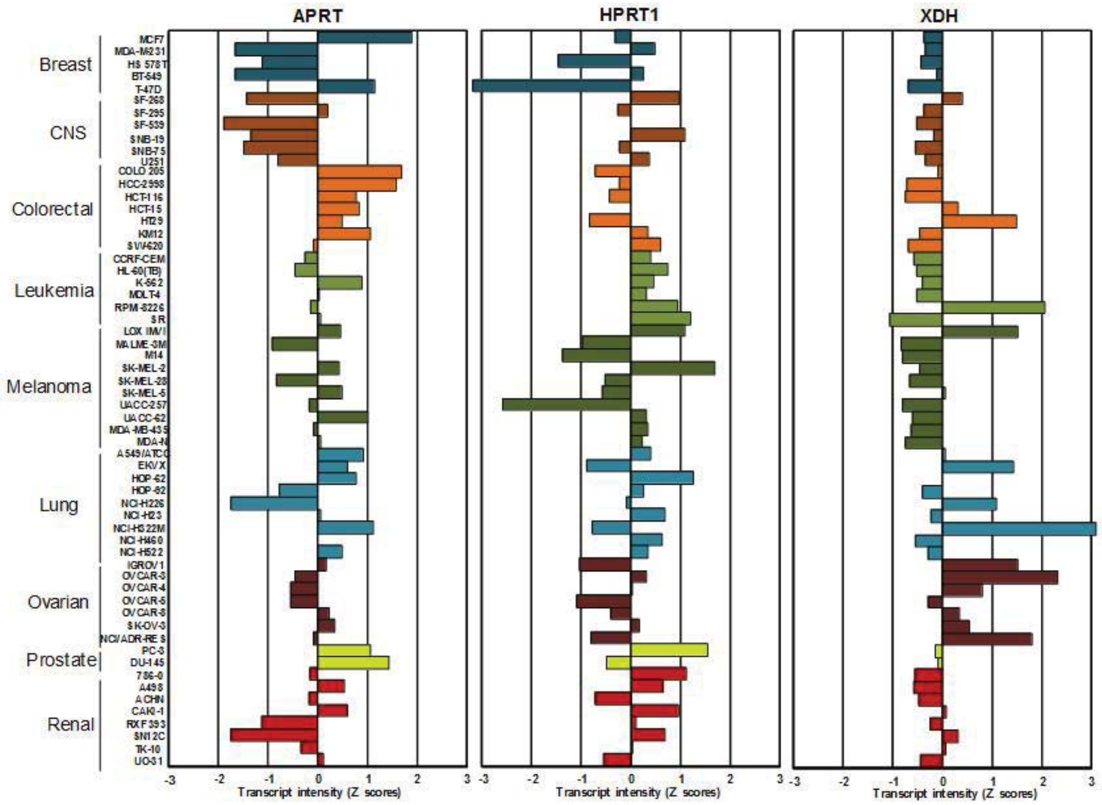

Suppl. Fig. 1: *APRT*, *HPRT1* or *XDH* expression in different cell lines of the NCI-60 panel. We present the transcript intensity for the *APRT*, *HPRT1* or *XDH* genes, expressed as z score.
